# Post-stroke delirium is a predictor of prolonged hospital stay and poor functional outcome at 3 months

**DOI:** 10.3389/fstro.2025.1719748

**Published:** 2026-01-15

**Authors:** Yacine Boudiba, Robin Gens, Anissa Ourtani, Gaël De Backer, Kaat Guldolf, Fenne Vandervorst, Sylvie De Raedt

**Affiliations:** 1 Department of Neurology, Universitair Ziekenhuis Brussel (UZ Brussel), Brussels, Belgium; 2 Neuroprotection and Neuromodulation (NEUR) Research Group, Center for Neurosciences (C4N), Vrije Universiteit Brussel (VUB), Brussels, Belgium; 3 Department of Neurology, Centre Hospitalier Universitaire Brugmann (CHU Brugmann), Brussels, Belgium; 4 Faculty of Medicine and Pharmacy, Vrije Universiteit Brussel, Brussels, Belgium; 5 Department of Neurology, Ziekenhuis aan de Stroom (ZAS) Middelheim and Hoge Beuken, Antwerpen, Belgium

**Keywords:** acute ischemic stroke, length of hospital stay, mortality, outcome, post-stroke delirium

## Abstract

**Background:**

Delirium is a frequent complication of acute ischemic stroke associated with poor outcome. The complex interplay with post-stroke infections remains to be elucidated. Our study aimed to investigate whether post-stroke delirium (PSD) was a predictor of prolonged hospital stay, poor functional outcome, and mortality after acute ischemic stroke, independent of the development of post-stroke pneumonia (PSP) and post-stroke urinary tract infections (PSU).

**Methods:**

In a previously published dataset of 514 patients with acute ischemic stroke, 201 patients (39%) developed delirium within the first week after stroke onset using a chart review method based on the Diagnostic and Statistical Manual of Mental Disorders, 5th Edition criteria. Fifteen percent developed PSP and 22% PSU, using the modified criteria of the US Centers for Disease Control and Prevention. Logistic regression analyses were used to identify predictors of prolonged hospital stay (>median 9 days), poor functional outcome (modified Rankin Scale >2), and mortality at 3 months after stroke onset.

**Results:**

Multiple logistic regression analysis showed that PSD was a predictor of prolonged hospital stay [odds ratio (OR): 4.085, 95% confidence interval (CI): 2.445–6.824] and poor functional outcome [OR: 3.362, 95% CI: 1.851–6.107) at 3 months after stroke onset, even after adjustment for age, premorbid disability, National Institutes of Health Stroke Scale on admission, PSP, and PSU. PSD was no predictor of mortality after stroke.

**Conclusion:**

PSD is a predictor of prolonged hospital stay and poor functional outcome at 3 months after ischemic stroke, independent of PSP and PSU.

## 1 Introduction

Delirium is a neuropsychiatric syndrome characterized by an acute state of confusion, typically fluctuating over the course of a day (American Psychiatric Association, 2013). It is very common in many healthcare settings with a prevalence of 11%−42% in acute medical hospital admissions (Siddiqi et al., 2006). Delirium is often seen as a marker of underlying pathology, an epiphenomenon, and the diagnosis is frequently missed. However, delirium is independently associated with adverse outcomes, including prolonged hospitalization, increased post-discharge mortality, institutionalization, and dementia (Siddiqi et al., 2006; Witlox et al., 2010).

Delirium affects one in four acute stroke patients with rates varying between 7 and 50% (Shaw et al., 2019). Growing evidence suggests a similar pattern of increased mortality and poor outcome after post-stroke delirium (PSD) (Shaw et al., 2019; Qu et al., 2018; Shi et al., 2012; Fialho Silva et al., 2021; Dostovic et al., 2016). Infections are also common complications after stroke, with a prevalence of up to 30%, one-third consisting of post-stroke pneumonia (PSP), and another third of post-stroke urinary tract infections (PSU). PSP in particular has been linked to poor stroke outcome (Westendorp et al., 2011). PSP and PSU are also both related to PSD (Guldolf et al., 2021).

We aimed to investigate whether delirium was a predictor of prolonged hospital stay, unfavorable functional outcome, and mortality at 3 months after ischemic stroke, independent of the development of pneumonia and urinary tract infection.

## 2 Materials and methods

### 2.1 Patients and assessment procedures

**Figure 1:**
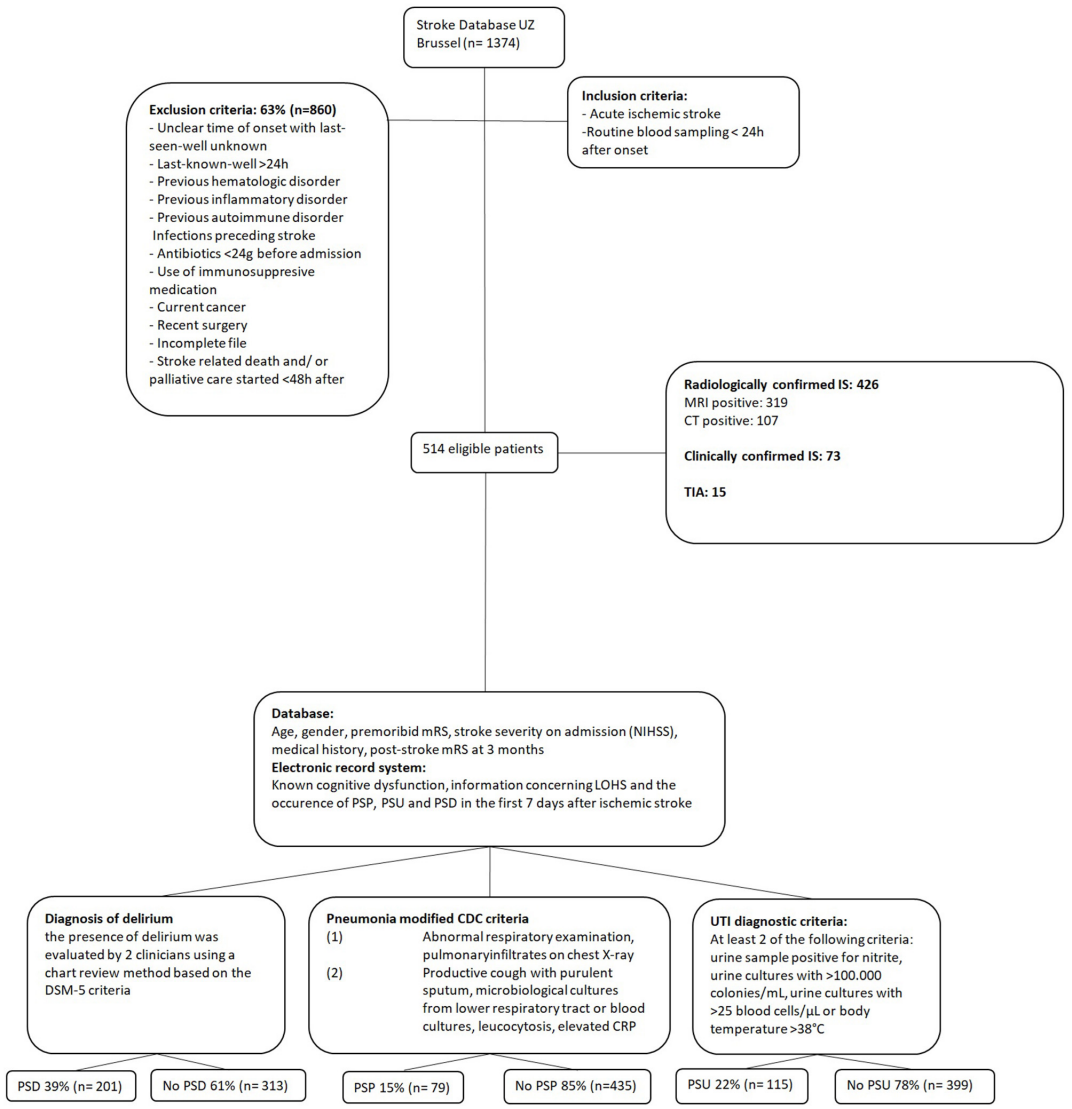
Study population flowchart. IS, ischemic stroke; mRS, modified Rankin Scale; NIHSS, National Institutes of Health Stroke Scale; LOHS, length of hospital stay; PSP, post-stroke pneumonia; PSU, post-stroke urinary tract infection; PSD, post-stroke delirium; DSM, Diagnostic and Statistical Manual of Mental Disorders; CDC, Centers for Disease Control and Prevention; CRP, C-reactive protein; UTI, urinary tract infection.

The current study is a secondary analysis of published data. The aim of the original study was to investigate predictors of post-stroke complications, such as PSD and post-stroke infections (pneumonia and urinary tract infection), with a focus on the predictive role of the neutrophil-to-lymphocyte ratio (Guldolf et al., 2021). We reviewed prospectively collected data of patients admitted to the Stroke Unit of the University Hospital Brussels (Belgium) over a 6-year period. We included all patients with acute ischemic stroke (AIS) who presented within 24 h after symptom onset. Exclusion criteria were unclear time of onset with last seen well unknown, previous hematologic, inflammatory or autoimmune disorders, infections preceding stroke, use of antibiotics less than 24 h before admission, use of immunosuppressants on admission, current cancer, recent surgery, incomplete file, and stroke-related death and/or palliative care started < 48 h after stroke onset (Figure 1). Demographic data, medical history, premorbid modified Rankin scale (mRS), National Institutes of Health Stroke Scale (NIHSS) on admission, and mRS at 3 months after stroke were retrieved from the database.

We also collected the length of hospital stay (LOHS) and information on the presence of premorbid cognitive dysfunction (premorbid cognitive impairment or dementia, e.g., diagnosis made at a memory clinic) after examination of the medical, nursing, and social staff notes.

The occurrence of PSD, based on the Diagnostic and Statistical Manual of Mental Disorders, Fifth Edition (DSM-5) criteria, as well as the occurrence of PSP and PSU using the modified criteria of the US Centers for Disease Control and Prevention, during the first 7 days after AIS (Guldolf et al., 2021) was evaluated based on medical and nursing records in our electronic records system.

Urinary tract infection was defined as the presence of at least two of the following four criteria: a nitrite-positive urine sample, a urine culture showing >100,000 colonies/mL, a urine culture containing >25 white blood cells/μL, or a body temperature >38 °C.

Diagnosis of pneumonia required fulfillment of at least one criterion from each of the following groups: (A) abnormal respiratory examination or pulmonary infiltrates on chest X-ray; (B) productive cough with purulent sputum, microbiological cultures from the lower respiratory tract or blood cultures, leukocytosis, or elevated C-reactive protein (CRP).

Delirium cases were identified through retrospective chart review by screening all medical and nursing records of the first 7 days of hospitalization on the presence of all five criteria of DSM-5 for delirium, resulting in a clinical vignette for each patient (Tables 1, 2). Two independent raters assessed each case. If they disagreed or if one rater was unable to decide, a third rater was consulted [which occurred in 94 of 514 cases (18%)]. The evaluation team consisted of one stroke neurologist (SDR) and two last-year neurology residents with subspecialty interest in stroke (FV) and neurodegenerative disorders (KG). More details are provided in previous publications (Guldolf et al., 2021). The study protocol was approved by the Ethics Committee of the University Hospital of Brussels (reference number B.U.N. 143201733949).

**Table 1:** Univariate analyses for prolonged hospital stay, unfavorable functional outcome, and mortality at 3 months.

**Variables**	**Prolonged LOHS (*****n*** = **514)**			**Unfavorable outcome at 3 months (*****n*** = **504)**			**Mortality at 3 months (*****n*** = **504)**			**Whole population**
	**Yes (*****n*** = **236)**	**No (*****n*** = **278)**	***p*** **-value**	**Yes (*****n*** = **231)**	**No (*****n*** = **273)**	***p*** **-value**	**Dead (*****n*** = **67)**	**Alive (*****n*** = **437)**	***p*** **-value**	
Age, years^†^	78 (68–85)	72 (60–81)	< 0.001	80 (72–87)	69 (58–78)	< 0.001	82 (74–88)	74 (62–83)	< 0.001	75 (63–83)
Gender, female^‡^	123 (52)	114 (41)	0.012	123 (53)	111 (41)	0.005	29 (43)	205 (47)	0.579	237 (46)
Premorbid mRS^†^	0 (0–1)	0 (0–0)	0.029	1 (0–3)	0 (0–0)	< 0.001	1 (0–3)	0 (0–0)	< 0.001	0 (0–1)
Premorbid mRS > 2^‡^	26 (12)	27 (10)	0.496	52 (26)	1 (0.4)	< 0.001	16 (28.)	37 (9)	< 0.001	53 (11)
NIHSS, adm^†^	11 (5–17)	3 (2–9)	< 0.001	13 (7–18)	3 (1–7)	< 0.001	15 (10–21)	5 (2–12)	< 0.001	7 (2–14)
Stroke subtype			0.004			0.837			0.817	
Radiologically confirmed IS	208 (88)	218 (78)		190 (82)	226 (83)		57 (85)	359 (82)		426 (83)
Clinically confirmed IS	26 (11)	47 (17)		33 (14)	40 (15)		8 (12)	65 (15)		73 (14)
TIA	2 (1)	13 (5)		8 (4)	7 (3)		2 (3.0)	13 (3.0)		15 (3)
PSP^‡^	61 (25.8)	18 (6.5)	< 0.001	69 (30)	10 (4)	< 0.001	37 (55)	42 (10)	< 0.001	79 (15)
PSU^‡^	84 (36)	31 (11)	< 0.001	79 (34)	32 (12)	< 0.001	23 (34)	88 (20)	0.009	115 (22)
PSD^‡^	145 (61	56 (20)	< 0.001	157 (68)	41 (15)	< 0.001	49 (731)	149 (34)	< 0.001	201 (39)
LOHS^†^	15 (12–20)	6 (5–8)	< 0.001	13 (9–18)	7 (5–10)	< 0.001	11 (7–18)	9 (6–14)	0.016	9 (6–15)
Premorbid cognitive dysfunction^‡^	28 (12)	26 (9)	0.355	48 (21)	5 (12)	< 0.001	15 (22)	38 (9)	< 0.001	54 (10)

Baseline characteristics of patients with vs. without long length of hospital stay (n = 514), unfavorable outcome (n = 500) as well as mortality (n = 500) at 3 months after ischemic stroke. Results are expressed as median [interquartile range (IQR)] or n (%) when appropriate.

LOHS, length of hospital stay; mRS, modified Rankin Scale; NIHSS, National Institutes of Health Stroke Scale; PSD, post-stroke delirium; PSP, post-stroke pneumonia; PSU, post-stroke urinary tract infection.

^†^Mann–Whitney U-test.

^‡χ2^ test.

**Table 2:** Stepwise MLRA predictors of prolonged length of hospital stay (>9 days).

**Variables**	**OR**	**95% CI**	***p*-value**
Gender, female	1.679	1.086–2.595	0.020
Premorbid disability	0.792	0.637–0.984	0.035
Ischemic stroke vs. TIA	5.743	1.140–28.937	0.034
NIHSS on admission	1.074	1.040–1.109	< 0.001
PSD	5.362	3.274–8.781	< 0.001
**After enrollment of PSP and PSU**			
Premorbid disability	0.743	0.598–0.924	0.008
NIHSS on admission	1.065	1.030–1.102	< 0.001
PSD	4.085	2.445–6.824	< 0.001
PSP	1.991	0.996–3.979	0.051
PSU	3.918	2.243–6.842	< 0.001

MLRA, multiple logistic regression analysis; OR, odds ratio; CI, confidence interval; NIHSS, National Institutes of Health Stroke Scale; PSD, post-stroke delirium; PSP, post-stroke pneumonia; PSU, post-stroke urinary tract infection.

### 2.2 Statistics

Statistical analyses were performed using the SPSS version 29.0 software package. Patients were compared based on short vs. long LOHS, favorable vs. unfavorable functional outcome, and dead vs. alive at 3 months. Median LOHS was used as cut-off for short vs. long hospital stay. Unfavorable functional outcome was defined as having a mRS > 2 (a cut-off commonly used in stroke research to separate patients being dependent or dead from those who remain functionally independent) (Saver et al., 2021). Normality was checked using the Kolmogorov-Smirnov test as well as visual interpretation of histograms and Q–Q plots. The Independent-Samples Student's *t*-test and the Mann–Whitney *U*-test were used for continuous variables, and the χ^2^ or Fisher exact test was used for categorical variables. Multiple logistic regression analysis (MLRA, backward: Wald) was used to identify independent predictors of long hospital stay, poor functional outcome, and mortality. For each of these outcome parameters, two MLRAs were performed to create two different models. For the first model, all variables enrolled without PSP and PSU, whereas in the second model, PSP and PSU were also added to the analysis.

## 3 Results

### 3.1 Baseline characteristics

In total, 514 patients were included in our final analysis. We identified three stroke subtypes: 426 patients had a radiologically confirmed stroke (319 on MRI, 107 on CT), 73 patients had a clinical diagnosis of ischemic stroke (acute onset of neurological deficit lasting >24 h; patients with no follow-up imaging after admission CT or a negative MRI/CT at follow-up), and 15 patients were diagnosed with a transient ischemic attack (TIA) (acute onset of neurological deficit lasting < 24 h, no other cause on brain CT/MRI). Of all patients included, median age was 75 (63–83). There were slightly fewer female patients included (46.1%), and the median stroke severity, measured through NIHSS, was 7 [interquartile range (IQR) 2–14]. Median LOHS was 9.0 (6.0–15.0) days, with 45.9% (*n* = 236/514) of patients having a long hospital stay (>9 days). Data on mRS at 3 months were missing in 10 patients. Of all patients, 45.8% (*n* = 231/504) suffered an unfavorable outcome (mRS > 2) at 3 months after ischemic stroke. Mortality at 3 months was 13.3% (*n* = 67/504).

### 3.2 Outcome after ischemic stroke–univariate analyses

Univariate analyses for prolonged hospital stay, unfavorable functional outcome, and mortality at 3 months are summarized in Table 1. Our results indicate that age, female gender, premorbid disability (mRS), stroke subtype, stroke severity on admission (NIHSS), and occurrence of PSP, PSU, and PSD were all associated with a prolonged hospital stay. Unfavorable functional outcome was associated with all these factors, except for stroke subtype, and with the addition of a known premorbid cognitive dysfunction. Similar results were found for mortality, except for gender, which did not seem to affect mortality in our study population.

### 3.3 Outcome after ischemic stroke–multiple logistic regression analyses

#### 3.3.1 Post-stroke delirium is a predictor of length of hospital stay and unfavorable outcome, also after adjustment for PSP and PSU

MLRA was used to identify independent predictors of prolonged LOHS. The median length of hospital stay, which was 9 days, was used as a cutoff for short vs. long LOHS. To identify predictors of prolonged LOHS, the following variables were enrolled in a first model: age, gender, premorbid mRS, stroke subtype, stroke severity at admission (NIHSS), and PSD. PSP and PSU were added to these variables in a second model. MLRA showed that PSD was a predictor of prolonged LOHS in both models, meaning it was independent of the occurrence of PSP and PSU, both of which were also predictors of prolonged LOHS (Table 2).

To identify predictors of poor functional outcome, only patients with a premorbid mRS ≤ 2 (88.7%, *n* = 417/470) were included. The following variables were enrolled in a first model: age, gender, premorbid mRS, known premorbid cognitive dysfunction, stroke severity at admission (NIHSS), and PSD.

PSP and PSU were added to these variables in a second model. PSD is a predictor of unfavorable functional outcome, and remained so, as well as PSP, in the second model (Table 3). PSU was not associated with poor functional outcome.

**Table 3:** Stepwise MLRA–predictors of unfavorable outcome at 3 months after exclusion of patients with a premorbid mRS > 2.

**Variables**	**OR**	**95% CI**	***p*-value**
Age	1.041	1.018–1.064	< 0.001
Premorbid disability	2.099	1.290–3.416	0.003
NIHSS on admission	1.171	1.122–1.222	< 0.001
PSD	4.080	2.306–7.220	< 0.001
**After enrollment of PSP and PSU**			
Age	1.043	1.019–1.068	< 0.001
Premorbid disability	2.633	1.572–4.410	< 0.001
NIHSS on admission	1.166	1.115–1.220	< 0.001
PSD	3.362	1.851–6.107	< 0.001
PSP	6.946	2.674–18.043	< 0.001

Stepwise MLRA for unfavorable outcome at 3 months after ischemic stroke.

MLRA, multiple logistic regression analysis; mRS, modified Rankin scale; OR, odds ratio; CI, confidence interval; NIHSS, National Institutes of Health Stroke Scale; PSD, post-stroke delirium; PSP, post-stroke pneumonia; PSU, post-stroke urinary tract infection.

#### 3.3.2 Post-stroke delirium does not predict mortality

In a first MLRA to identify predictors of mortality, we used the following variables: age, gender, premorbid mRS, known cognitive dysfunction, stroke severity (NIHSS), and PSD. Subsequently, PSP and PSU were again added to the analyses. PSD was not associated with mortality in either model. However, age, gender, premorbid disability (mRS), stroke severity on admission (NIHSS), and PSP were predictors of mortality (Table 4).

**Table 4:** Stepwise MLRA–predictors of mortality at 3 months.

**Variables**	**OR**	**95% CI**	***p*-value**
Age	1.042	1.011–1.074	0.008
Gender, female	0.352	0.175–0.709	0.003
Premorbid disability	1.524	1.185–1.960	0.001
NIHSS on admission	1.144	1.095–1.194	< 0.001
**After enrollment of PSP and PSU**			
Age	1.039	1.006–1.074	0.022
Gender, female	0.515	0.242–1.096	0.085
Premorbid disability	1.613	1.230–2.115	< 0.001
NIHSS on admission	1.104	1.054–1.156	< 0.001
PSP	7.682	3.645–16.188	< 0.001

Stepwise MLRA for mortality at 3 months after ischemic stroke.

MLRA, multiple logistic regression analysis; OR, odds ratio; CI, confidence interval; NIHSS, National Institutes of Health Stroke Scale; PSP, post-stroke pneumonia; PSU, post-stroke urinary tract infection.

## 4 Discussion

The main finding of our study is that PSD is a predictor of prolonged hospital stay and poor functional outcome at 3 months, but not of mortality, independent of the development of PSP and PSU, the two most common infectious complications after AIS.

Most earlier studies had already reported that PSD was a predictor of prolonged hospital stay (Saver et al., 2021; Nydahl et al., 2017). Only Nydahl and coworkers found no association between PSD and LOHS (Nydahl et al., 2017). A partial explanation of this finding could be the inclusion of TIA patients. Most studies included mixed stroke subtypes (both ischemic and hemorrhagic) and reported only univariate analysis (Saver et al., 2021; Nydahl et al., 2017; Klimiec-Moskal et al., 2019; Oldenbeuving et al., 2011; Sheng et al., 2006). Oldenbeuving and coworkers reported a prolonged hospital stay (5.4 days longer) after adjustment for age, stroke severity, and premorbid cognitive function [Informant Questionnaire on Cognitive Decline in the Elderly (IQCODE) score]. The additional value of our study is that we adjusted for age, gender, premorbid mRS, stroke severity, and, especially, PSP and PSU and that we precluded hemorrhagic stroke.

Our study also demonstrated an association between PSD and poor functional outcome at 3 months after stroke, consistent with the majority of previous research (Fialho Silva et al., 2021).

Most of the studies evaluated here included patients with mixed stroke subtypes, with the exception of Lim et al. (2017), who focused on ischemic stroke but did not adjust for potential confounders.

Two of the more recent studies also adjusted for post-stroke infections (Fialho Silva et al., 2021), PSP and PSU (Klimiec-Moskal et al., 2019).

Qu and coworkers showed an association between PSD and functional outcome as measured by Instrumental Activities of Daily Living, although this was not observed using mRS after adjustment for cofounders (Qu et al., 2018).

Oldenbeuving and coworker identified an association between PSD and the Barthel Index at 3 months, though functional outcome was not assessed using the mRS (Oldenbeuving et al., 2011).

Sheng and coworker also demonstrated an association between PSD and the Functional Independence Measure score at 12 months, but they did not evaluate functional outcome with the mRS (Sheng et al., 2006).

In our study, we found no association between PSD and mortality in multivariate analysis, including in the model with PSP and PSU. Previous studies obtained contradictory results. Some studies reported no association with early (in hospital) or late (6–12 months) mortality (Hénon et al., 1999), while others found a connection in univariate analyses but did not perform a multivariate analysis (McManus et al., 2009). Recently, a few studies did find PSD to be an independent predictor of mortality after controlling for factors like age, comorbidities, and stroke severity (Pasińska et al., 2019), although these findings are not consistent across all research (Rollo et al., 2022). Only one study adjusted for both pneumonia and urinary tract infection, despite analyzing a mixed population that included both ischemic and hemorrhagic strokes (Klimiec-Moskal et al., 2019). Variations in study populations and differences in methodology likely contribute to the ongoing uncertainty about whether PSD directly affects mortality after stroke

The strength of our study is that we studied three well-defined post-stroke complications, PSD, PSP, and PSU, within a single dataset. However, the study had several limitations. Since we used the retrospective chart review method for delirium based on DSM-5 criteria, we might have missed a couple of cases, especially for the hypoactive delirium subtype. We also did not study the delirium subtype of severity or duration. To reduce misdiagnosis, two independent evaluators reviewed independently all nursing and medical notes in the first 7 days of admission. In the first days, we had access to at least three (and often five or six) nursing notes in most patient files, including night-time notes, meaning good coverage of day and night periods. When no consensus was reached, a third independent evaluator was consulted. The same limitation is applicable to the retrospective diagnosis of PSP and PSU. However, the incidence of PSP, PSU (Westendorp et al., 2011), and PSD (Fleischmann et al., 2021) are comparable with those in prospective studies. Premorbid cognitive dysfunction was also determined retrospectively, and the 10% prevalence was likely an underestimate. Studies using the IQCODE suggest that about one-third of stroke patients have prestroke cognitive impairment (Mele et al., 2024). Another limitation of the study has to do with the extensiveness of the exclusion criteria (cf. flowchart) and the narrowness of the inclusion criteria (< 24 h after stroke onset). As a result, our findings might not be representative of the overall stroke population. In addition, we only adjusted for PSP and PSU, not for other infectious complications after stroke.

Other predictors of poor functional outcome and mortality at 3 months were age, stroke severity on admission (NIHSS), premorbid disability, and PSP. This is in line with the results of previous studies (Westendorp et al., 2011; Johnston et al., 2000).

PSU was only a predictor of prolonged hospital stay, but not of unfavorable outcome and mortality at 3 months. We speculate that this might be explained by the fact that, unlike pneumonia, most PSUs do not lead to systemic infections (Westendorp et al., 2022).

## 5 Conclusion

Our study demonstrates that PSD is a predictor of prolonged hospital stay and poor functional outcome at 3 months, independent of age, stroke severity, and the development of PSP and PSU, the two most frequent infectious complications in patients with AIS. This needs to be confirmed in prospective studies. Whether preventing PSD might improve stroke outcome will require further research.

## Data Availability

The raw data supporting the conclusions of this article will be made available by the authors, without undue reservation.
